# Glucose control of root growth direction in *Arabidopsis thaliana*

**DOI:** 10.1093/jxb/eru146

**Published:** 2014-04-09

**Authors:** Manjul Singh, Aditi Gupta, Ashverya Laxmi

**Affiliations:** National Institute of Plant Genome Research, Aruna Asaf Ali Marg, New Delhi 110067, India

**Keywords:** *Arabidopsis*, brassinosteroid, endocytosis, glucose.

## Abstract

Directional growth of roots is a complex process that is modulated by various environmental signals. This work shows that presence of glucose (Glc) in the medium also extensively modulated seedling root growth direction. Glc modulation of root growth direction was dramatically enhanced by simultaneous brassinosteroid (BR) application. Glc enhanced BR receptor BRASSINOSTEROID INSENSITIVE1 (BRI1) endocytosis from plasma membrane to early endosomes. Glc-induced root deviation was highly enhanced in a PP2A-defective mutant, *roots curl in naphthyl phthalamic acid 1-1* (*rcn1-1*) suggesting that there is a role of phosphatase in Glc-induced root-growth deviation. RCN1, therefore, acted as a link between Glc and the BR-signalling pathway. Polar auxin transport worked further downstream to BR in controlling Glc-induced root deviation response. Glc also affected other root directional responses such as root waving and coiling leading to altered root architecture. High light intensity mimicked the Glc-induced changes in root architecture that were highly reduced in Glc-signalling mutants. Thus, under natural environmental conditions, changing light flux in the environment may lead to enhanced Glc production/response and is a way to manipulate root architecture for optimized development via integrating several extrinsic and intrinsic signalling cues.

## Introduction

The ability for plant organs to guide their growth at a specified angle from the gravity vector (gravitropism) ensures that the shoot is positioned to maximize its light-harvesting capabilities and that the roots are positioned downward so as to maximize the uptake of water and nutrients. The ability to readjust their growth direction by sensing any deflection relative to the direction of gravity involves gravity perception and signal transduction, followed by differential growth (Morita, 2010).

The differential growth response with respect to gravity is supported by the role of phytohormones as mediator to coordinate the process (Philosoph-Hadas *et al.*, 2005). Auxin signalling and distribution is the most widely studied phenomenon in response to root gravitropism. Differential auxin accumulation leads to asymmetric cell elongation causing roots to curve. PIN-FORMED (PIN) proteins, which recycle via clathrin-mediated endocytosis (Kitakura *et al.*, 2011), determine auxin flow in response to gravity (Rakusová *et al.*, 2011). Cytokinin also plays a regulatory role in root gravitropism. Exogenous cytokinin applied to vertical roots induced root bending towards the application site, confirming the inhibitory effect of cytokinin in root gravitropism (Aloni *et al.*, 2004). Ethylene negatively regulates root gravitropism in an ETHYLENE RESISTANT1 (ETR1) and ETHYLENE INSENSITIVE2 (EIN2) -dependent manner; ethylene inhibits gravity response by altering flavonoid synthesis (Buer *et al.*, 2006). Recent reports have also shown that abscisic acid (ABA) acts as a negative regulator of gravitropic response in *Arabidopsis* roots (Han *et al.*, 2009). The negative effect of ABA is a result of change in ionic strength and NO_3_ signal (Han *et al.*, 2009). In another study, a tryptophan amide-linked conjugate of jasmonic acid has been shown to cause agravitropic root in *Arabidopsis* (Staswick, 2009). Gibberellic acid (GA) has also been shown to modulate root curvature. Inhibitory effect of GA on PIN protein trafficking downstream to brefeldin A (BFA)-sensitive endosomes causes stabilization of PIN2 protein at the lower side of root, thus changing auxin flow and redistribution required for gravitropic bending (Löfke *et al.*, 2013).

Brassinosteroid (BR) is important modulator of plant growth; BRs promote organ growth by affecting cell elongation and cell division. The effects of BR on root gravitropic response have been studied. Exogenous BL application has been shown to increase the gravitropic curvature in primary roots (Kim *et al.*, 2000). BRs increase auxin polar transport in the root via increasing the activity of RHO-RELATED PROTEIN FROM PLANTS 2 (ROP2), subsequently enhancing gravitropic response of *Arabidopsis* roots (Li *et al.*, 2005). Low levels of indole-3-acetic acid increases BR-mediated root gravitropism and vice versa in *Arabidopsis* (Kim *et al.*, 2007). BR also affects actin filament configuration and PIN2 localization similar to that of auxin thus affecting tropism (Lanza *et al.*, 2012).

Sugars are best known as metabolic substrates but also have an important signalling function (Price *et al.*, 2004). It has been shown that along with root growth and development in general, glucose (Glc) can also influence root directional responses via modulating auxin response pathway (Mishra *et al.*, 2009). BR and Glc have also been shown to modulate hypocotyl directional growth in *Arabidopsis* (Gupta *et al.*, 2012). Previous studies have provided significant evidence of interactions between sugar and phytohormone response and other metabolic pathways (Gibson, 2004; Ramon *et al.*, 2008). Altogether, there are a number of reports that individually focus on the role of different hormones or sugars in controlling *Arabidopsis* root directional responses.

The work provides an insight for Glc regulation of BR signalling via modulating BRI1 endocytosis, following a series of hierarchical signalling events, to alter the root growth direction.

## Materials and methods

### Plant materials

*Arabidopsis thaliana* ecotypes of Col-0, Ws, Ler, and En-2 were used as wild-type controls. Seeds of *gin2-1*, *rgs1-1*, *rgs1-2*, *gpa1-1*, *gpa1-2*, *gpa1-3*, *thf1-1*, *bri1-6*, *bzr1-1D*, *rcn1-1*, *eir1-1*, and *aux1-7* were obtained from the ABRC (http://www.arabidopsis.org/abrc/). Following lines were obtained from the original published source as: pBRI1::BRI1::GFP (Friedrichsen *et al.*, 2000), 35S::GFP-ABD2-GFP (Wang *et al.*, 2008), and *mdr1-1* (AT3G28860; Noh *et al.*, 2001). All mutant lines were in Col background except the following: the *bri1-6* mutant was in the En-2 background; *gpa1-1*, *gpa1-2*, *rcn1-1*, and *mdr1-1* were derived from Ws background; and *gin2-1* was in the Ler background.

### Growth conditions

Seeds were surface sterilized and imbibed at 4 °C for 48h. The imbibed seeds were germinated and grown vertically on Petri dishes containing 1/2 MS supplemented with 1% sucrose (Suc) and solidified with 0.8% agar. Seed germination was carried out in climate-controlled growth rooms under a 16/8 light/dark cycle (22±2 °C and light intensity 60 μmol m^–2^ s^–1^). For experiments testing the effects of different sugars/hormones/inhibitors on root directional growth, 5-d-old light-grown seedlings were transferred to the treatment medium and kept vertically under culture room conditions. For experiments exploring the root directional growth on increasing light intensity, light intensities ≈2000 Lux (≈30 μmol m^–2^ s^–1^) and ≈7000 Lux (≈100 μmol m^–2^ s^–1^) were used. Root coiling and waving responses were studied as described previously (Buer *et al.*, 2003; Chen *et al.*, 2009). For root coiling, imbibed seeds were germinated and grown on a horizontally placed media for 7 d. To study root waving, imbibed seeds were germinated on a hard agar (1.5%) media surface and grown vertically for 48h, then tilted at 45° for 5 d. All chemicals were purchased and prepared as described previously (Gupta *et al.*, 2012). Brassinazole (BRZ), staurosporine, K252a, okadaic acid, cantharidin, latrunculin B (Lat-b), cycloheximide (CHX), and BFA (Sigma) were prepared as 10^–2^ M stock solutions and FM4-64 (Invitrogen) was prepared as a 2mM stock solution in dimethyl sulphoxide.

### Measurements

For each experiment, 5-d-old uniformly grown seedlings were transferred to treatment mediums and positions of their root tips were marked at the back sides of plates. Digital images of seedlings were captured using Nikon Coolpix digital camera after 3 d. For gravitropic-bending kinetics, pictures were taken at different time intervals after gravistimulation. The root deviation was measured by calculating the angle of root from the vertical axis using ImageJ (http://rsb.info.nih.gov/ij/). Root elongation was measured using ImageJ. Root coiling and waving response was studied as described previously (Buer *et al.*, 2003; Chen *et al.*, 2009). Digital images were captured and processed using ImageJ. For biomass, 5-d-old uniformly grown seedlings were transferred to treatment medium for 10 d, root and shoot tissues were harvested, and fresh weight was measured. The area spanned by roots was measured using ImageJ.

### Laser confocal scanning microscopy

To determine the BRI1 receptor endocytosis in root, 5-d-old light-grown pBRI1::BRI1::GFP-expressing seedlings were transferred on treatment medium for 24h. Confocal images of the root tip epidermal cells were captured using a laser confocal scanning microscope (TCS SP5 AOBS, Leica Microsystems, Heidelberg, Germany). For imaging GFP, the 488nm line of the Argon laser was used for excitation and emission was detected at 520nm. For imaging FM4-64, 543nm line of the helium/neon laser was used for excitation and emission was detected at 590–620nm. All fluorescence intensity measurements were performed using LCS Lite software version 2.61 (Leica Microsystems). GFP/FM4-64 experiments were performed by using sequential scanning. To observe the actin filament organization in root epidermal cells, 5-d-old light-grown, 35S::GFP-ABD2-GFP-expressing seedlings were transferred on treatment medium for 24h. The laser, pinhole, and gain settings of the confocal microscope were kept identical for different treatments. Images were assembled using Photoshop (Adobe Systems). At least three biological replicates, with each replicate having 10 seedlings, were performed for all the experiments.

### Cell patterning in root epidermis and amyloplast staining

Five-d-old light-grown Col-0 (WT) seedlings were transferred to Glc-free or 3% (w/v) Glc-containing 1/2 MS medium solidified with 0.8% agar in a climate-controlled growth room for 3 d under a 16/8 light/dark cycle. The root epidermal cell profile visualization and amyloplast staining was performed as previously described for etiolated hypocotyls in Gupta *et al.*, 2012.

### Gene expression analysis

For quantitative real-time PCR analysis, *Arabidopsis thaliana* Col-0 seeds, 5 d after germination on 1/2 MS medium supplemented with 0.8% agar and 1% Suc, were transferred to Glc-free and 3% Glc-containing medium for 48h. Root tissue from each sample was harvested in liquid N_2_ and processed for RNA isolation. Total RNA was extracted and QC was performed as described (Gupta *et al.*, 2012). Two biological replicates with three technical replicates each were used. For each gene tested, expression values in control sample (0% Glc) were taken as absolute and the values in treated samples (3% Glc) were normalized against untreated samples (0% Glc). The primer sequences for all the genes tested are given in Supplementary Table S1 (available at *JXB* online).

### Statistics

All experiments reported in this work were performed at least three times yielding similar results. Each experiment was considered as an independent biological replicate. All data measures are averages from two independent biological replicates each with at least 20 seedlings otherwise specified. Error bars represent SE. Statistical significance for all the experiments was evaluated using Excel (Microsoft). For all experiments, statistical differences between both control/treatment and WT/mutant pairs were analysed using Student’s t-test evaluation with paired two-tailed distribution. A cut off for *P* value was 0.001 except where stated otherwise. All endpoint analyses were taken 72h after transfer to treatment medium otherwise specified, although plates were observed for up to 10 d.

## Results

### Glc induces root growth direction changes independently of root length changes

**Fig. 1.**
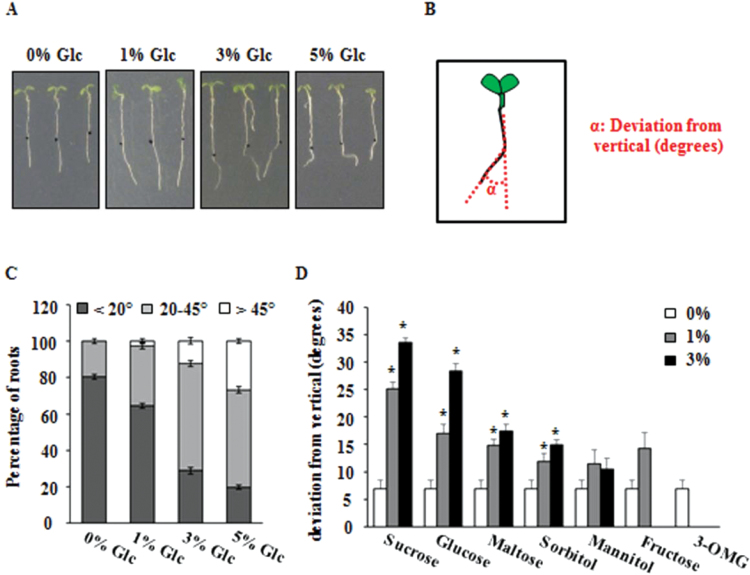
Exogenous glucose (Glc) induces root deviation from vertical in *Arabidopsis*. (A) Root deviation from vertical of WT (Col-0) at different Glc concentrations. (B) Method of quantification of the primary root deviation angle from vertical gravity vector (α). (C) Degrees of deviation from vertical at different Glc concentrations after 72h. (D) Root deviation from vertical at indicated concentrations of various sugar analogues after 72h. Besides sucrose and Glc, only maltose and sorbitol caused some root deviation from vertical, but to a very little extent. 3-OMG, 3-*O*-methyl-d-glucopyranose. Data are mean±SE of two biological replicates with at least 30 seedlings. Asterisks indicate significant differences (*P*<0.001, Student’s t-test): * control vs. treatment.

WT (Col-0) seeds were germinated and grown vertically on increasing concentrations of Glc-containing 1/2 MS medium solidified with 0.8% agar in the light. The primary root grew vertically down in Glc-free medium, whereas on increasing concentrations of Glc, the primary root showed deviation from straight/vertical growth in a dose-dependent manner (Fig. 1A). Root deviation relative to gravity vector was measured as angle of deflection after 72h of transfer to the treatment media (Fig. 1B, C). To check if this root directional response was a direct effect of light, the Glc-induced root deviation was studied in darkness. Five-d-old light-grown WT seedlings were transferred to Glc-free or 3% Glc-containing 1/2 MS media and the plates were then kept in dark for 3 d. Essentially, Glc was able to cause root direction reset in dark (Supplementary Fig. S1A). Study of root elongation during the course of deviation suggests that changes in root length do not correspond to extent of root deviation. The roots of 5% Glc-grown seedlings were the smallest, yet they showed the highest degree of deviation as compared to seedling roots growing on 0, 1, and 3% Glc (Fig. 1A).

Root gravitropism kinetics of gravistimulated seedlings in presence of Glc was also affected. Five-d-old WT (Col-0) seedlings grown on 1/2 MS media were transferred to different concentrations of Glc-containing media and subjected to gravity reorientation assay by giving a 90° gravistimulation. The root gravitropic response was quantified as a measurement of angle of curvature at various time points after the seedlings were reoriented. Higher Glc concentration (3%, 5% Glc) caused significant delay in gravitropic bending of roots at almost all time points tested (Supplementary Fig. S1B). The maximum gravitropic curvature angle recorded for 5% Glc-treated seedling roots was ~50° at 72h after reorientation in contrast to the ~20° angle recorded for seedlings placed in Glc-free medium (Supplementary Fig. S1B). The root elongation kinetics during the course of gravistimulation suggests that rate of root elongation is independent of extent of root gravitropism in media containing different concentrations of Glc (Supplementary Fig. S1C).

### Root growth direction changes are induced by metabolizable sugars and are not merely an osmotic effect of sugars

In order to find if root deviation is Glc specific or other sugars could also induce similar response, the effect of various sugar analogues was observed. Five-d-old WT (Col-0) seedlings grown on 1/2 MS media were transferred to different sugars and root deviation from vertical was checked after 72h. The seedlings could show pronounced root deviation from vertical on Suc- and Glc-containing media whereas other slowly metabolizable sugars displayed the response at a much lesser extent (Fig. 1D). The main endogenous sugar found in the apoplast is Suc. Therefore, the pronounced directional response on Suc media could be due to the better efficiency of Suc in entering tissues than Glc, through active uptake into cells, or that Suc is further degraded into Glc and fructose monomers after uptake (Sairanen *et al.*, 2012). The osmotic sugars mannitol and sorbitol could show very little response, suggesting that osmotic changes in the medium are not solely responsible for the observed response. 3-*O*-Methyl-d-glucopyranose (3-OMG), which is transported to cell but not sensed as a sugar, has been used as Glc analogue to study sugar signalling (Jang and Sheen, 1994; Martin *et al.*, 1997; Ho *et al.*, 2001; Cortès *et al.*, 2003). In the current study, 3-OMG could not support root growth and deviation (Fig. 1D and Supplementary Fig. S2A).

### Glc-induced root growth deviation does not involve amyloplast degradation

Amyloplasts play a significant role in gravity sensing and are essential for full gravitropic response (Kiss *et al.*, 1989). Both salt stress and root hydrotropic stress have been reported to degrade amyloplast to cause root growth deviation (Takahashi *et al.*, 2003; Sun *et al.*, 2008). To investigate whether Glc could also cause root deviation by degrading amyloplasts, the roots were stained using I_2_-KI solution. There was no disruption of amyloplasts in columella cells in fact, an increase in amyloplasts was observed at higher concentration of Glc (Supplementary Fig. S2B) suggesting that Glc-mediated gravitropic defect may not involve amyloplast degradation as a way to induce root deviation.

### Glc-induced root growth direction changes involve both hexokinase-dependent and -independent signal transduction pathways

The Glc-signalling pathways in *Arabidopsis* includes AtHXK1 (HEXOKINASE1)-dependent and -independent components. In the AtHXK1-dependent pathway, HXK1 acts as a Glc sensor (Jang *et al.*, 1997; Moore *et al.*, 2003). We have previously shown that the *gin2* mutant exhibits significantly enhanced Glc-induced root deviation response (Mishra *et al.*, 2009). The AtHXK1-independent pathway involves the G-protein signalling component (Assmann 2002; Jones 2002; Chen and Jones, 2004; Chen *et al.* 2006; Temple and Jones 2007; Urano *et al.*, 2013). To further elucidate the role of HXK1-independent Glc-signalling components in Glc-mediated root directional changes, root deviation response in *rgs1-1*, *rgs1-2*, *thf1-1*, *gpa1-1*, *gpa1-2*, and *gpa1-3* mutant seedlings was measured. While *rgs1-1*, *rgs1-2*, and *thf1-1* showed a significantly reduced Glc-induced root deviation from vertical, *gpa1-1*, *gpa1-2*, and *gpa1-3* displayed significantly higher degree of root deviation from vertical at all Glc concentrations tested (Supplementary Fig. S2C). Root growth measurement showed no correlation between the effects of Glc on root deviation angle and root elongation in HXK1-independent Glc-signalling mutants (Supplementary Fig. S2D). These results suggest that Glc involves both HXK-dependent and -independent signal transduction pathways to modulate root directional growth.

Gravitropic bending kinetics was also assayed in gravitationally stimulated seedlings of the Glc-signalling mutants *gin2-1* and *rgs1-1*. The *gin2* mutant exhibited significant decrease in gravitropic-bending kinetics (Supplementary Fig. S3A) while *rgs1-1* showed slightly increased gravitropic-bending kinetics as WT (Supplementary Fig. S3B). These results further substantiated the involvement of both HXK-dependent and -independent signal transduction in Glc modulation of root directional responses.

### Glc involves BR-signalling elements for inducing root growth deviation

**Fig. 2.**
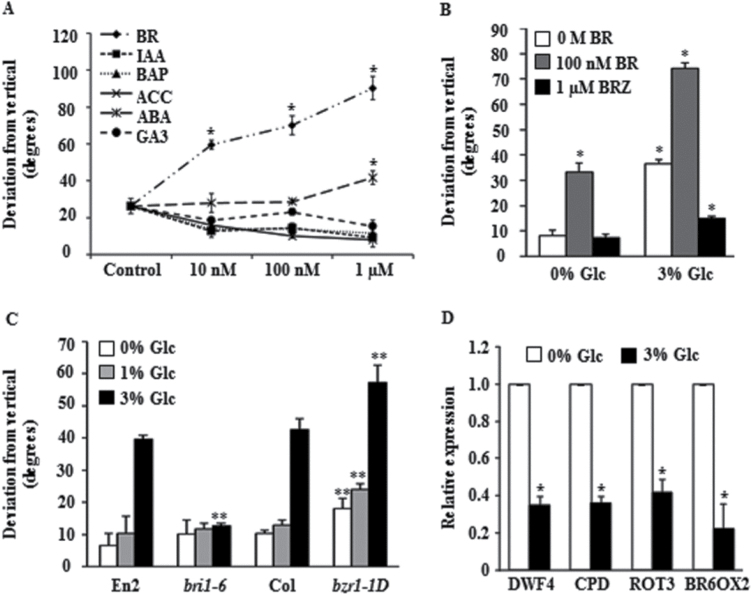
Involvement of BR signalling in controlling Glc-induced root deviation from vertical. (A) Effect of different phytohormones on WT seedling roots, to determine their roles in root deviation response; BR even at a very low concentration (10nM) exerted root directional defects, whereas ABA at a higher concentration (1 μM) produced some effect. (B) Glc-induced root deviation from vertical in 5-d-old WT (Col-0) seedlings in absence or presence of BR and BRZ; BRZ treatment completely abolished Glc-induced root deviation. (C) Glc-induced root deviation from vertical in the BR-perception mutant *bri1-6* and the BR-signalling mutant *bzr1-1D* at different Glc concentrations. (D) Real-time expression analysis of BR-biosynthesis genes upon Glc treatment; reduced expression of BR-biosynthesis genes indicated enhanced BR signalling. ACC, 1-aminocyclopropane-carboxylic acid hydrochloride; BAP, 6-benzylaminopurine; BR, 24-epibrassinolide; BR6OX2, *BRASSINOSTEROID-6-OXIDASE 2*; BRZ, brassinazole; CPD, *CONSTITUTIVE PHOTOMORPHOGENESIS AND DWARFISM*; DWF4, *DWARF 4*; GA3, gibberellic acid; IAA, indole acetic acid; ROT3, *ROTUNDIFOLIA 3*. Expression values in controls were taken as absolute and values in treated samples were normalized against untreated samples. Data are mean±SE of two biological replicates with at least 30 seedlings. Asterisks indicate significant differences (*P*<0.001, Student’s t-test): * control vs. treatment; ** WT vs. mutant).

Plant hormones such as auxin, cytokinin, jasmonic acid, ABA, and BR have been shown to play important roles in affecting root directionality (Aloni *et al.*, 2004; Li *et al.*, 2005; Han *et al.*, 2009; Staswick, 2009; Strohm *et al.*, 2012). To find out if Glc signalling is mediated by different phytohormone, effect of different hormones was tested in inducing root growth deviation. Five-d-old light-grown WT seedlings transferred on IAA, BAP, ACC, or GA3 -containing 1/2 MS medium did not show any root directional defects. Only 24-epibrassinolide (BR) could influence the root growth direction in a dose-dependent manner. ABA could also induce a small amount of root deviation but at a very high concentration (Fig. 2A). Presence of BR along with Glc synergistically induces root growth deviation from vertical. Inhibition of BR biosynthesis using BRZ could strongly reduce Glc-mediated root growth deviation (Fig. 2B and Supplementary Fig. S4B). All these results in combination suggest that Glc may involve downstream BR signalling in controlling Glc-induced root growth deviation.

To find out the involvement of different BR-signalling components in Glc-induced root deviation response, the deviation of BR-signalling mutants was checked in presence of Glc. Compared to WT, the *brassinosteroid insensitive1-6* (*bri1-6*) mutant, which lacks the reception ability towards BR, showed no root growth deviation in presence of Glc (Fig. 2C and Supplementary Fig. S4D). Roots of the BR-hyperresponsive mutant *brassinazole resistant1-1D* (*bzr1-1D*) displayed an exaggerated Glc-mediated root deviation response as compared to WT (Fig. 2C and Supplementary Fig. S4D), further confirming that BR signalling positively mediates Glc-induced root deviation. Root growth measurement showed no correlation between root deviation and root elongation (Supplementary Fig. S4A, C, E).

It has been reported that enhanced BR signalling reduces expression of BR biosynthesis genes (Wu *et al.*, 2011). To further confirm if Glc could actually enhance BR signalling, the transcript levels of the BR-biosynthetic genes *DWF4*, *CPD*, *ROT3*, and *BR6OX2* were studied in presence of Glc (Fig. 2D). Five-d-old light-grown WT seedlings were transferred to either Glc-free or 3% Glc-containing 1/2 MS media for 48h and gene expression studies were performed with excised root tissues. The expression of tested BR biosynthetic genes was reduced in Glc-treated roots, indirectly suggesting that Glc may enhance BR signalling and in turn can reduce the expression of BR biosynthetic genes through feedback mechanisms.

### Glc induces BR-receptor BRI1 endocytosis in the roots

**Fig. 3.**
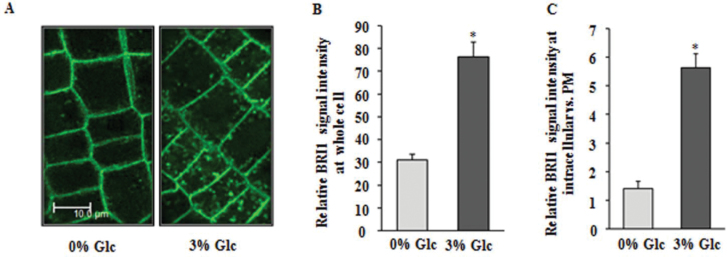
Glc increases BR signalling via enhancing BRI1 endocytosis. (A) BRI1::GFP fluorescence in 5-d-old light-grown seedling root tip treated with 3% Glc for 24h; All confocal images were generated by using 63X objective, bar=10.0 μm. (B) BRI1 abundance at the whole-cell level in control and 3% Glc-treated seedling roots. (C) Relative BRI1-GFP abundance at intracellular versus plasma membrane signal in epidermal cells of pBRI1::BRI1::GFP-expressing seedling roots; in Glc-free medium, there was uniform BRI1 localization to plasma membrane with very small internalization, whereas in 3% Glc-treated roots, there was significantly higher BRI1 internalization to endosomal compartments. Data are mean±SE of three biological replicates with at least 10 roots, with 10 cells counted per root. Asterisks indicate significant differences (*P*<0.001, Student’s t-test): * control vs. treatment.

To find out how Glc could enhance BR signalling, the effect of increasing concentrations of Glc was observed on pBRI1::BRI1::GFP line. It was found that in light-grown *Arabidopsis* root epidermal cells, Glc treatment led to enhanced accumulation of BRI1 protein in the early endocytic vesicles, thus leading to a pronounced occurrence of BRI1-GFP in endosomes (Fig. 3A). The early endosome marker FM-464 was used to colocalize BRI1::GFP (Supplementary Fig. S4F). Glc not only increased the overall BRI1 abundance in cell, but also significantly increased the ratio between intracellular versus plasma-membrane-localized BRI1 signal (Fig. 3B, C). It has been reported that increase in the ratio of endosomal to plasma-membrane BRI1 signal can enhance downstream BR-signalling events (Russinova *et al.*, 2004; Geldner *et al.*, 2007). The Glc-induced internalization of BRI1::GFP suggest that Glc may enhance BR signalling via enhancing BRI1 endocytosis/internalization.

### Glc may enhance BRI1 internalization via affecting protein phosphatase activity

**Fig. 4.**
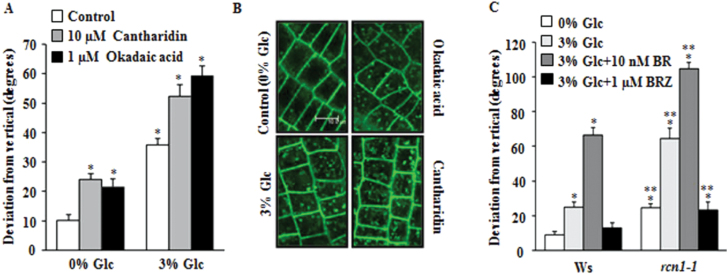
Glc may alter root growth direction by reducing protein phosphatase 2A activity. (A) Root directional growth in WT (Col-0) seedlings upon 10 μM cantharidin and 1 μM okadaic acid treatment in presence and absence of Glc. (B) BRI1::GFP localization upon cantharidin and okadaic acid treatments; inhibition of protein phosphatase activity led to enhanced BRI1 internalization even in absence of Glc. (C) Root directional growth of *rcn1-1* and WT in presence or absence of 3% Glc and/or BR and BRZ after 72h; confocal images were generated using 63X objective, bar=10.0 μm. BR, 24-epibrassinolide; BRZ, brassinazole. Data are mean±SE of two biological replicates with at least 20 seedlings. Asterisks indicate significant differences (*P*<0.001, Student’s t-test): * control vs. treatment; ** WT vs. mutant).

To find out the exact mechanism of Glc regulation of BRI1 internalization, this work investigated the main components known to affect BRI1 endocytosis. The phenomenon of BRI1 internalization and recycling has been shown to be governed extensively by phosphorylation and dephosphorylation events (Di Rubbo *et al.*, 2011). In the present study, we used physiological treatments of known kinase and phosphatase inhibitors in presence of Glc to find the changes in root deviation. The protein kinase inhibitors staurosporin and K252a did not show any significant effect on Glc-induced root deviation (Supplementary Fig. S5A). The protein phosphatase inhibitors okadaic acid and cantharidin caused a pronounced increase in Glc-induced root deviation as compared to control (Fig. 4A and Supplementary Fig. S5B). Okadaic acid and cantharidin treatment could also enhance BRI1 endocytosis similar to Glc supplementation (Fig. 4B). Previous studies have indicated the involvement of variety of protein phosphatase 1 (PP1) and 2A (PP2A) in plant sugar signalling (Ramon *et al.*, 2008). To establish a role of PP2A in Glc- and BR-induced root deviation response, we studied *ROOTS CURL IN NAPHTHYL PHTHALAMIC ACID1* (*RCN1*), which encodes one of the three members of regulatory subunit A and functions as a positive regulator of PP2A holoenzyme (Zhou *et al.*, 2004). The *rcn1-1* mutant has been linked to root gravitropism and the gravitropic defects of *rcn1-1* mutation are due to reduced PP2A activity (Rashotte *et al.*, 2001). In *rcn1-1*, BRI1 accumulation and BR signalling is increased (Wu *et al.*, 2011). To test whether Glc-mediated root deviation is due to its effect on PP2A activity, Glc sensitivity of *rcn1-1* was studied. In presence of Glc, the gravitropic defect of *rcn1-1* was enhanced, phenocopying to that of BR-treated seedlings, whereas normal gravitropic growth was restored upon BRZ treatment (Fig. 4C and Supplementary Fig. S5D). Root growth measurement showed no correlation between root deviation and root elongation (Supplementary Fig. S5C, E). Since BRI1 protein turnover in *Arabidopsis* has been shown to be regulated by activity of PP2A (Di Rubbo *et al.*, 2011), Glc probably alters the BR-signalling cascade via affecting RCN1.

To regulate signalling output, receptor internalization and its subsequent targeted fate such as recycling or degradation is very crucial. We also tried to test whether other inhibitors related to endocytosis/degradation can effect Glc-induced root deviation. Physiological treatments with the proteolysis inhibitor MG132 (Z-Leu-Leu-Leu-al), the protein-trafficking inhibitor BFA, and the protein biosynthesis inhibitor CHX, were used in presence of Glc to find the extent of the response. While CHX treatment inhibited overall growth of the seedlings, MG132 and BFA did not induce much difference as compared to WT (Supplementary Fig. S5F).

### Glc-induced root growth deviation may involve changes in polar auxin transport

**Fig. 5.**
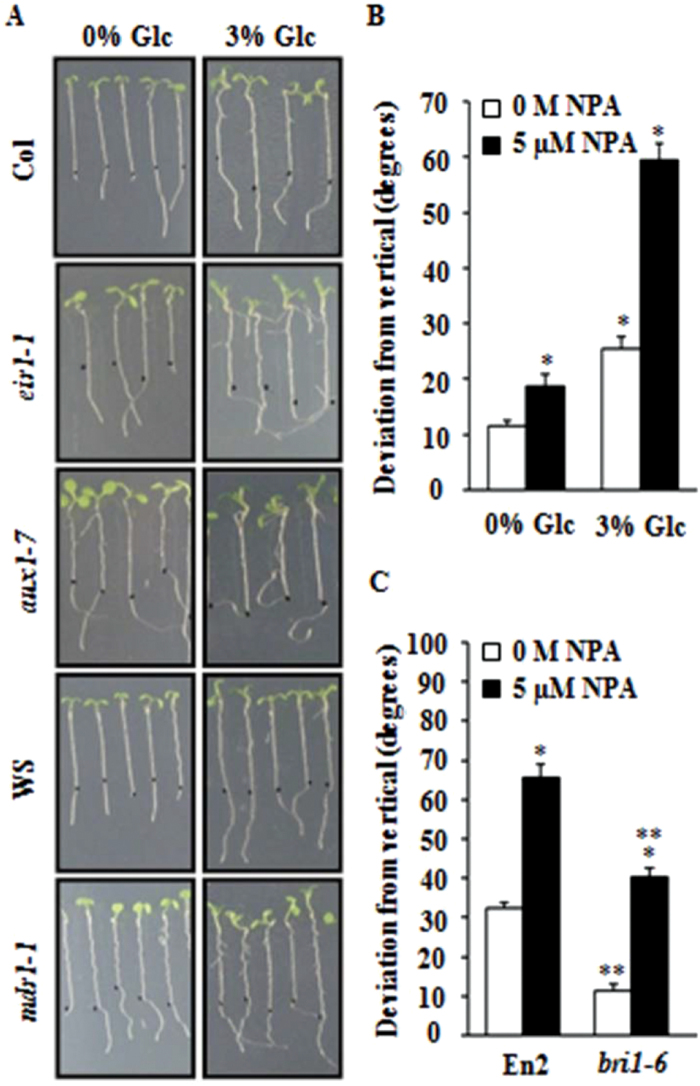
Involvement of auxin transport in Glc-induced root deviation response. (A) Glc-induced root deviation response in auxin transport-defective mutants; *eir1-1*, *aux1-7*, and *mdr1-1* displayed significantly enhanced root deviation response in presence of 3% Glc as compared with respective WT. (B) Effect of the polar auxin transport inhibitor NPA (5 μM) on root directional growth of WT (Col-0) seedlings in presence and absence of Glc; NPA treatment along with 3% Glc caused extensive root deviation. (C) NPA and 3% Glc induced root deviation from vertical in *bri1-6*, which is otherwise resistant towards Glc-induced root deviation. NPA, *N*-naphthyl phthalamic acid. Data are mean±SE of two biological replicates with at least 30 seedlings. Asterisks indicate significant differences (*P*<0.001, Student’s t-test): * control vs. treatment; ** WT vs. mutant).

Our group has previously reported that Glc-induced root growth deviation involves various auxin-signalling components (Mishra *et al.*, 2009). The involvement of auxin transport components in root gravitropism is also very well established (Luschnig *et al.*, 1998; Marchant *et al.*, 1999). The auxin polar transport mutants *ethylene insensitive root 1* (*eir1*) and *auxin resistant 1* (*aux1-7*) have been found to be involved in BR-mediated root gravitropism (Kim *et al.*, 2007). Mishra *et al.* (2009) showed that increasing Glc concentration could enhance basipetal auxin transport in WT roots. To further investigate which auxin transport components are involved in Glc-mediated root deviation, the effect of Glc was checked in different auxin polar transport-related mutants. The *eir1-1*, *aux1-7*, and *multidrug resistance 1* (*mdr1-1*) mutants showed exaggerated Glc-induced root growth deviation as compared to WT, suggesting that Glc may interact/modulate auxin transport machinery to cause Glc-induced root direction response (Fig. 5A). Auxin polar transport inhibitor *N*-naphthyl phthalamic acid (NPA) could also substantially enhance the Glc-induced root direction reset (Fig. 5B and Supplementary Fig. S6A), further supporting that Glc may modulate auxin transport machinery to cause Glc-induced root deviation. Auxin transport changes may work downstream even to BR response, since NPA was able to induce root deviation even in *bri1-6* (Fig. 5C and Supplementary Fig. S6C), which could not show Glc-induced root growth deviation. Root growth measurement showed no correlation between root deviation and root elongation (Supplementary Fig. S6B, D). All these observations suggest that differential auxin transport may lie downstream to Glc and BR in controlling root growth deviation.

### Glc-induced root deviation involves cytoskeletal reorganization and cell patterning

**Fig. 6.**
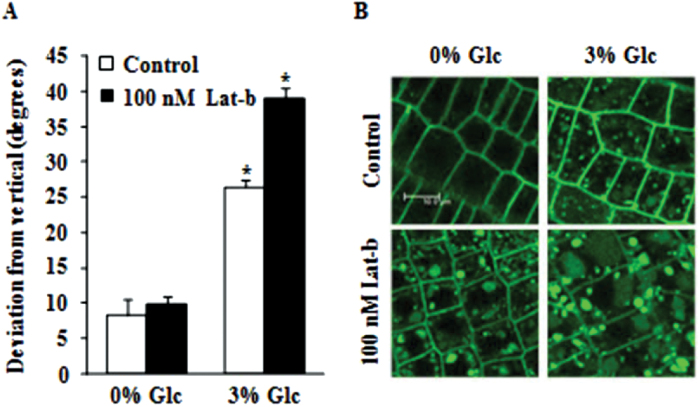
Involvement of cytoskeletal reorganization in Glc-induced root deviation. (A) Glc-induced root deviation from vertical in presence of the actin filament organization inhibitor Lat-b (100nM); Lat-b treatment with 3% Glc significantly enhanced the root deviation response. (B) BRI1::GFP localization upon Lat-b treatment; disruption of actin organization led to enhanced BRI1 internalization even in absence of Glc, and in presence of 3% Glc large intracellular bodies were formed; bar=10.0 μm. Lat-b, latrunculin B. Data are mean±SE of two biological replicates with at least 30 seedlings. Asterisks indicate significant differences (*P*<0.001, Student’s t-test): * control vs. treatment.

Cytoskeleton has been widely studied with respect to gravitropism, and reorganization of cytoskeleton has been shown to positively regulate gravitropism during the signal transduction stage and gravitropic bending. Glc has been reported to interfere with actin filament organization (Balasubramanian *et al.*, 2007). These findings prompted us to relate the Glc-induced root direction reset with actin cytoskeleton. Disrupting actin filament organization using Lat-b in presence of Glc could further enhance root deviation from vertical as compared to control (Fig. 6A). BRI1 internalization upon disruption of actin machinery using Lat-b was enhanced even in absence of Glc, and presence of Glc along with Lat-b could further cause formation of large intracellular bodies (Fig. 6B). Root growth measurement showed no correlation between root deviation and root elongation (Supplementary Fig. S6E). Exogenous Glc treatment also caused changes in the cell profile of the root epidermis, as seedlings grown in Glc-free 1/2 MS medium displayed a straight arrangement of epidermal cells across the root, while twisting of epidermal cells in a spiral manner was observed in Glc-treated roots (Supplementary Fig. S6F). Changes in actin filament organization in *Arabidopsis* roots upon Glc treatment was also studied using 35S::GFP-ABD2-GFP lines and it was found that Glc treatment caused alteration of actin filaments organization (Supplementary Fig. S6G).

### Adaptive significance of Glc-induced root growth deviation and architectural changes

**Fig. 7.**
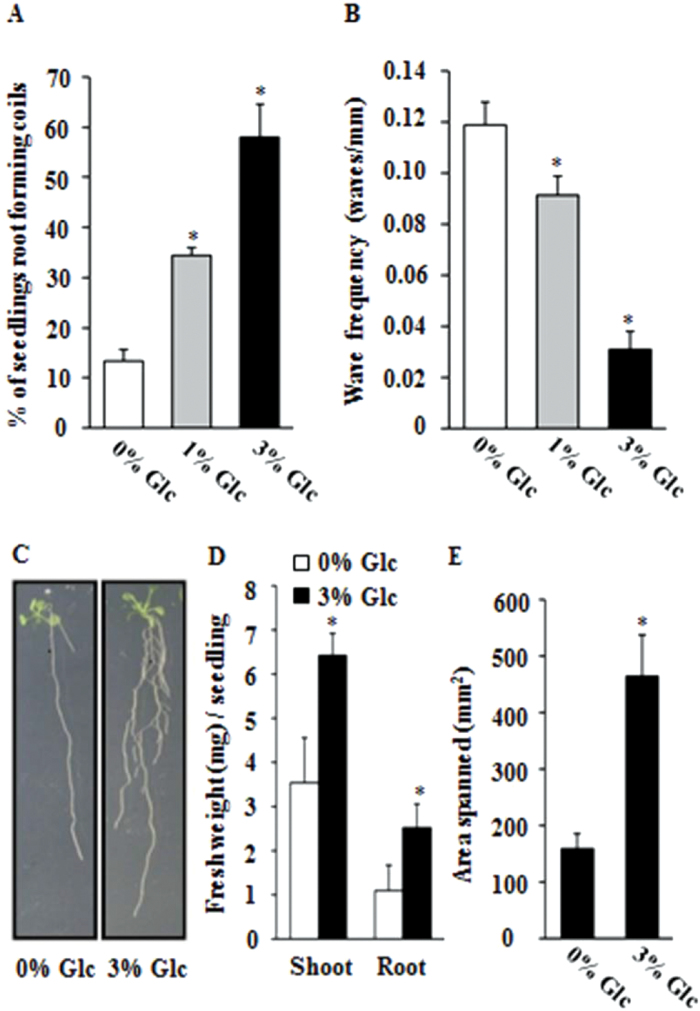
Glc triggers changes in other root directional responses and overall seedling architecture: quantitative assessment of the root-coiling and root waving phenotypes of WT (Col-0) seedlings without Glc or with increasing Glc concentration (1 and 3%). (A) Root coiling was significantly enhanced upon 3% Glc treatment. (B) Increased Glc concentration significantly inhibited spatial wave frequency of WT (Col-0) primary roots. (C) Effect of Glc on overall growth and architecture of early WT (Col-0) seedlings. (D) Higher Glc concentration significantly enhanced the biomass of roots as well as shoots. (E) Quantification of area spanned by the roots of 15-d-old WT (Col-0) seedlings on Glc-free or 3% Glc-containing media. Data are mean±SE of two biological replicates with at least 20 seedlings. Asterisks indicate significant differences (*P*<0.001, Student’s t-test): * control vs. treatment.

To find out the cumulative effect of Glc on root architecture, we studied the effect of Glc on root waving and coiling, the factors able to affect overall root architecture. Increasing Glc concentration increased the percentage of seedling roots that form coils (Fig. 7A and Supplementary Fig. S7A); the wave frequency was reduced with seedling roots forming larger waves (Fig. 7B and Supplementary Fig. S7B). Altogether, Glc-induced changes in root directional responses led to an increased area spanned by the seedling root, hence enabling better anchorage to the medium/soil and support for the enhanced corresponding shoot and root biomass (Fig. 7C–E).

### Role of Glc-induced root growth deviation under natural environmental conditions

**Fig. 8.**
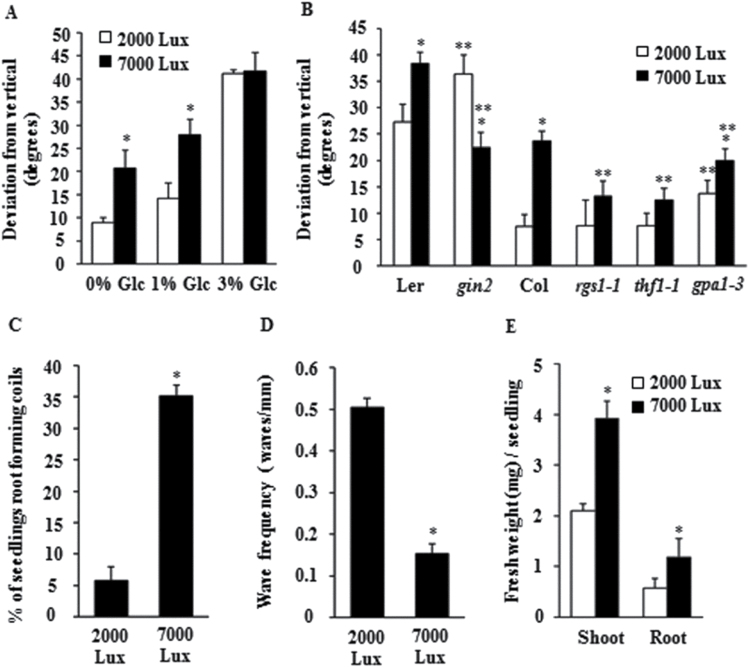
Photosynthetically generated sugar signals mimic exogenous Glc application effects on root directional responses as well as seedling architecture. (A) Glc-induced root deviation from vertical in WT (Col-0) seedlings at low (2000 Lux) and high (7000 Lux) light intensities. (B) High light flux induced root deviation from vertical in Glc-signalling mutants on Glc-free media; HXK1-dependent and -independent signalling mutants showed perturbed responses towards high light flux-induced root deviation. High light intensity mimicked the Glc effects on root coiling (C) and root waving (D) in WT (Col-0) seedling roots in Glc-free media. (E) Increased light flux increased root and shoot biomass in Glc-free media. Data are mean±SE of two biological replicates with at least 30 seedlings. Asterisks indicate significant differences (*P*<0.001, Student’s t-test): * control vs. treatment; ** WT vs. mutant).

The availability of Glc under natural environment conditions depends on the presence of light, and varying light conditions may modulate root architecture by using Glc as an intermediate. Light levels have been used in past to modulate endogenous sugar levels, which correlate positively with primary root growth and the density of lateral roots in *Arabidopsis* (Freixes *et al.*, 2002; Kircher and Schopfer, 2012). To test this hypothesis, we first checked whether light could also modulate root growth direction similar to that of Glc. Increasing light intensity from 2000 Lux to 7000 Lux could actually induce root growth deviation in 1/2 MS-grown WT (Col) seedlings, similar to Glc-induced root growth deviation (Fig. 8A and Supplementary Fig. S7C, D). To question whether a light signal is actually transduced by Glc, the effect of light on Glc-signalling mutants was studied in terms of root deviation. While *gin2* could not show light-induced root deviation from vertical, *rgs1-1, thf1-1*, and *gpa1-3* showed a significantly reduced sensitivity towards light-induced root deviation as compared to WT (Fig. 8B). Higher light intensity could also change root waving and coiling similarly to Glc-induced changes in root waving and coiling (Fig. 8C, D and Supplementary Fig. S7E, F), suggesting that Glc can mimic effect of light in controlling root architecture. Together, these results suggest that light may use Glc-signalling components to modulate root architecture under natural environmental conditions.

## Discussion

Plant root systems show high plasticity in development and can adapt their architecture in response to a variety of external stimuli to maintain optimal growth patterns (Lynch, 1995; Malamy, 2005). Plant roots need to overcome the signal from gravity and reorient their growth direction to navigate across or around physical obstacles or towards water and nutrients. Exposure to water or salt stress causes altered root gravitropic response by rapidly degrading amyloplasts in root columella cells of *Arabidopsis* (Takahashi *et al.*, 2003; Sun *et al.*, 2008). Mutants with reduced levels of amyloplast starch, such as *pgm* (*phosphoglucomutase*) and *eal1* (*endodermal-amyloplast less 1*), have also been reported to be less gravitropic (Kiss *et al.*, 1997; Fujihira *et al.*, 2000), suggesting an important role of amyloplast in controlling root direction. Here we report that metabolizable sugar, Glc, via hexokinase-dependent and -independent signalling, also modulated root growth direction of vertically grown seedlings and caused a significant decrease in bending of roots upon gravistimulation. Glc application led to a substantial increase in amyloplasts content in columella cells in contrast to water- and salt-stress-challenged roots, suggesting that Glc may not involve amyloplast degradation as a means of changing root growth directionality. The *Arabidopsis starch excess 1* (*sex1*) mutant, having greater amount of starch relative to the wild type, have also been shown to possess almost similar gravitropic sensitivity (Vitha *et al.*, 2007), substantiating the current conclusion.

Glc-induced root deviation was highly enhanced by simultaneous BR application, suggesting an important role of BR in controlling root directional growth. In fact, Glc treatment led to enhanced accumulation of BR receptor BRI1 protein in the early endocytic vesicles. Endosomal trafficking from plasma membrane to endocytic compartments has been suggested as being very important for gravitropic response (Strohm *et al.*, 2012). Enhanced BRI1 accumulation in the endocytic vesicle may eventually lead to enhanced BR signalling. In fact, increase in the ratio of endosomal to plasma membrane BRI1 signal has been reported to enhance downstream BR-signalling events (Geldner *et al.*, 2007). This assumption is further strengthened by the fact that Glc led to downregulation of BR biosynthetic genes, which indirectly suggests that BR signalling is more in Glc-treated seedling roots. It has been reported that enhanced BR signalling leads to downregulation of BR biosynthesis genes (Wu *et al.*, 2011). The enhanced BR signalling may eventually regulate root directionality by previously known mechanisms such as modulation of auxin transport or actin filament organization (Lanza *et al.*, 2012).

Endosomal trafficking from plasma membrane to endocytic compartments has been suggested as being very important for gravitropic response (Strohm *et al.*, 2012). BRI1 protein homeostasis in *Arabidopsis* is regulated by activity of protein phosphatases PP2A (Di Rubbo *et al.*, 2011). Phosphatase inhibitor treatments alone could enhance BRI1-GFP endocytosis. The *rcn1-1* mutant, which is defective in protein phosphatase activity, shows more BRI1 accumulation in endocytic vesicles and enhanced BR signalling (Wu *et al.*, 2011). Glc-mediated root deviation was also found to be enhanced in *rcn1-1*. The enhanced Glc-induced root deviation could be reverted back by BR biosynthesis inhibitor BRZ, further suggesting that the involvement of enhanced BR signalling is responsible for Glc-induced changes in root growth direction. RCN1, therefore, acts as a link between Glc and the BR-signalling pathway, and Glc probably via affecting RCN1 alters the BR-signalling cascade in regulating root directional response.

There are previous reports that BR signalling may modulate gravitropism via altering auxin polar transport (Li *et al.*, 2005). We did observe that Glc-induced root directional changes were dramatically enhanced in auxin polar transport mutants. Auxin polar transport inhibitor NPA could induce root growth directional changes, even in *bri1-6*, which is resistant to Glc-induced root directional changes. suggesting that auxin transport lies downstream to BR.

Cytoskeleton reorganization has been widely studied with respect to control of gravitropic responses (Rahman *et al.*, 2007; Nick *et al.*, 2009). Both auxin and BR have been shown to be involved in cytoskeleton rearrangement and cellular patterning (Dhonukshe *et al.*, 2008; Lanza *et al.*, 2012). Presence of Glc also alters actin filament organization and cellular patterning (Balasubramanian *et al.*, 2007; Supplementary Fig. S6F, G). These results in combination suggest that Glc, either directly or via modulating BR and auxin response, can alter the cytoskeleton and cellular patterning the most downstream steps responsible for inducing Glc-induced root deviation.

### A testable model for Glc-induced root deviation response

**Fig. 9.**
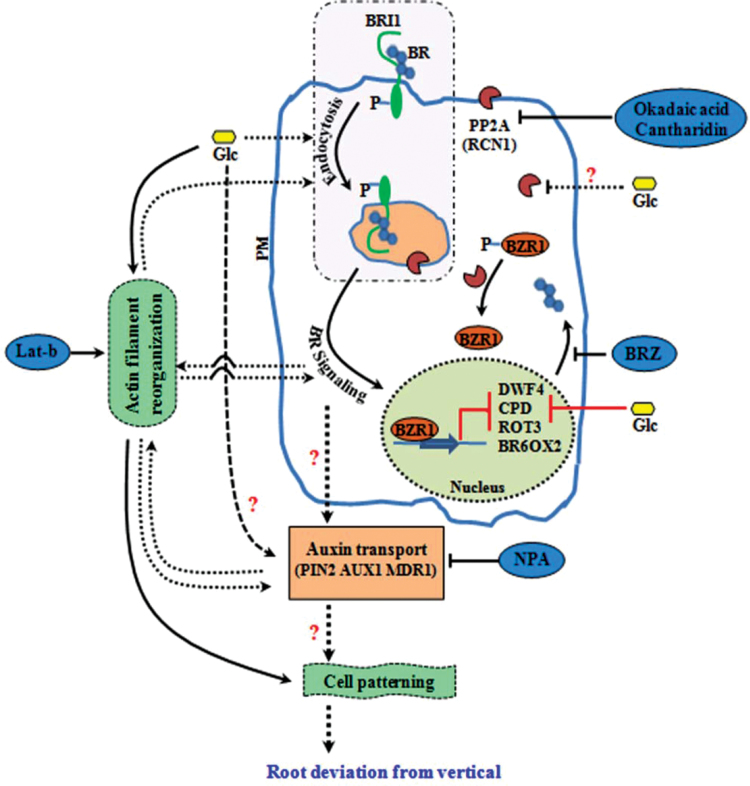
A testable model for Glc-induced root deviation response based on the present findings and previous reports.

A testable model based upon the aforementioned findings and previously published reports is presented in Fig. 9. Glc causes root deviation from vertical by using both hexokinase-dependent and -independent Glc-signalling pathways (Mishra *et al.*, 2009; Fig. 1 and Supplementary Figs S2C and S3). Glc enhanced BR signalling to induce root deviation response by increasing BR receptor BRI1 internalization (Figs 2, 3 and Supplementary Fig. S4). Increased BRI1 endocytosis upon Glc treatment may be a results of reduced PP2A activity, as phosphatase inhibitors okadaic acid as well as cantharidin also caused increased BRI1 endocytosis and root deviation similar to Glc treatment (Fig. 4A, B). *rcn1-1* also showed exaggerated Glc-induced root deviation that could be restored by application of BRZ (Fig. 4C). RCN1, therefore, acts as a link between Glc and the BR-signalling pathway. These results also suggest that auxin may work further downstream to BR, since auxin transport inhibitor NPA treatment along with Glc caused root deviation in *bri1-6*, which was otherwise insensitive to Glc-induced root deviation (Fig. 5C). Also, the auxin transport mutants *eir1-1, aux1-7*, and *mdr1-1* displayed highly exaggerated Glc-induced root deviation response (Fig. 5A). Increasing Glc application, either directly or via altered BR and auxin response, could translate to changes in root growth direction. This model may provide a foundation for testing and for investigation of additional routes available for root deviation from vertical response.

### Adaptive significance of the response under natural environmental conditions

Root growth direction can be correlated with architectural stability to provide increased anchorage and water and nutrient absorption. Increasing Glc concentration not only induced root deviation from vertical but also altered root waving and coiling, eventually leading to a root architecture that was able to better support the aboveground parts. Glc treatment also increased the area spanned by seedling root for better exploration of the soil for water and nutrient. All these features in combination would lead to a root architecture leading to better fitness in a natural environment condition. Root architecture has been reported to be involved in regulating seedling fitness and survival (Chen *et al.*, 2009).

Under natural environmental conditions, Glc as such is not available to the plant root. Plants can produce Suc via photosynthesis in the presence of light, which is eventually hydrolysed to Glc and fructose, the only source of Glc to plant root. Thus, availability of Glc under natural environment conditions depends on the presence of light, since high light intensity boosts photosynthesis, sugar production, and Glc signalling in a physiological context in plants (Moore *et al.*, 2003; Xiong *et al.*, 2013). The hypothesis in this case is that varying light conditions may modulate root architecture by using Glc as an intermediate. The hypothesis is supported by the fact that increasing light intensity could actually induce root growth deviation similar to Glc-induced root growth deviation. Higher light intensity could also change root waving and coiling similar to Glc-induced changes in root waving and coiling. Light-induced changes in root architecture were significantly curtailed in the Glc-signalling mutant. Together, these results suggest that light may use Glc-signalling components to modulate root architecture under natural environmental conditions. Recently, the role of light-generated Suc has been shown to be involved in regulating root elongation (Kircher and Schopfer, 2012). Photosynthesis-derived Glc in shoot drives target-of-rapamycin (TOR) signalling relays through glycolysis and mitochondrial bioenergetics to control root meristem activation and in turn root growth (Xiong *et al.*, 2013). Here, we are proposing the involvement of Glc and downstream hormones in regulating root directional responses eventually leading to optimal root architecture under changing light conditions. Further investigation of hierarchical events involved in this directional response may lead us to better understand the phenomenon of root architecture modulation during early seedling development in natural environment.

## Supplementary material

Supplementary data are available at *JXB* online.

Supplementary Fig S1. Glc-induced root deviation from vertical is not correlated with root growth inhibition.

Supplementary Fig. S2. Role of Glc metabolism and signalling in root growth and direction.

Supplementary Fig. S3. Both HXK -dependent and -independent components of Glc signalling are involved in root deviation from vertical response.

Supplementary Fig. S4. Involvement of BR-signalling elements in regulating root directional growth.

Supplementary Fig. S5. Involvement of protein phosphorylation and de-phosphorylation in regulating root directional response.

Supplementary Fig. S6. Changes in polar auxin transport and actin cytoskeleton organization regulate Glc-induced root directional response.

Supplementary Fig. S7. High light intensity could mimic Glc effects on root waving and coiling responses.

Supplementary Table S1. List of primers used for real-time PCR analysis.

Supplementary Data
