# Supplementary material for: Glucose control of root growth direction in Arabidopsis thaliana
Source: J Exp Bot. 2014 Apr 9;65(12):2981–93. doi: 10.1093/jxb/eru146 (PMC4071822; doi:10.1093/jxb/eru146)
Supplement: Supplementary Data [file supp_65_12_2981__index.html]

Glucose control of root growth direction in Arabidopsis thaliana — Supplementary Data 

# Glucose control of root growth direction in *Arabidopsis thaliana*

## Supplementary Data

Data files

**Files in this Data Supplement:**

- Supplementary Data - Supplementary Data
