# Supplementary material for: Glucose control of root growth direction in Arabidopsis thaliana
Source: J Exp Bot. 2014 Apr 9;65(12):2981–93. doi: 10.1093/jxb/eru146 (PMC4071822; doi:10.1093/jxb/eru146)
Supplement: Supplementary Data [file supp_eru146_supplementary_figures_13_3_14.pdf]

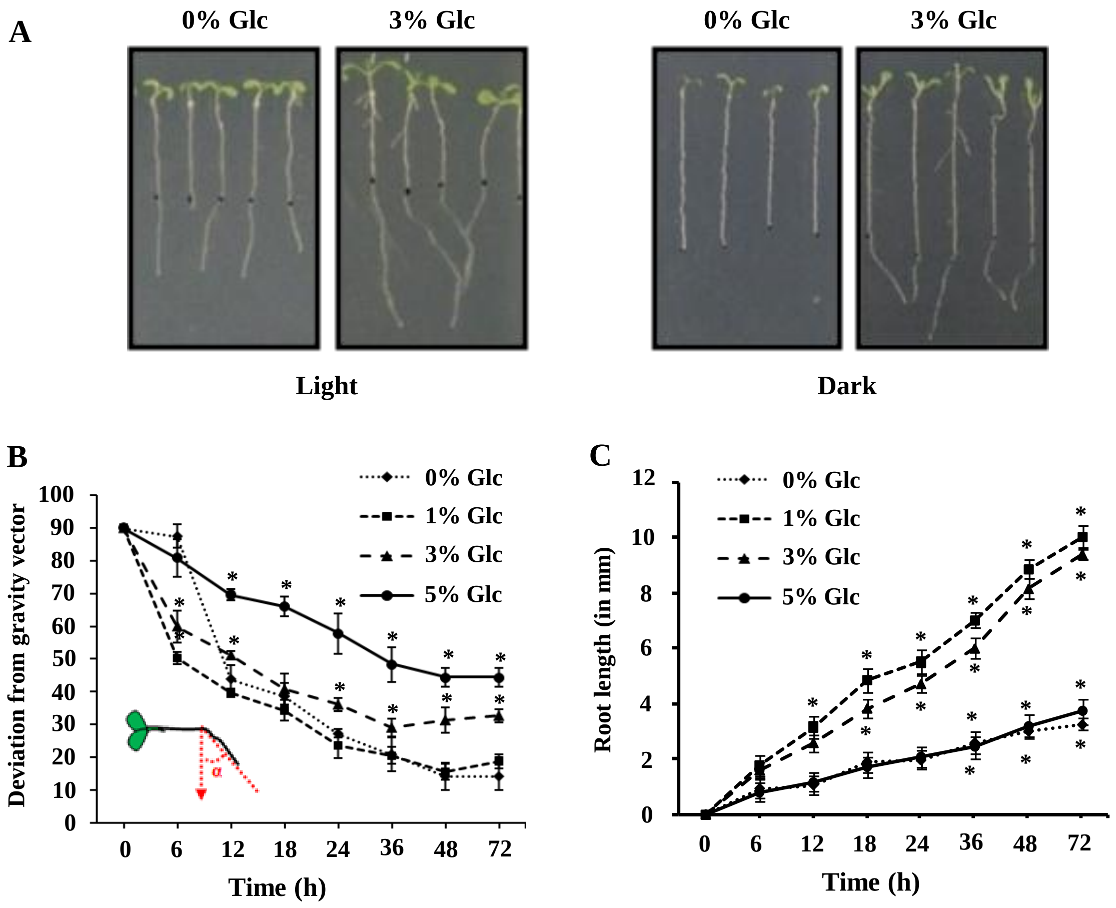

Supplementary figure. S1. Glc induced root deviation from vertical is not correlated with root growth inhibition.

A, Glc induced root directional reset under light and dark conditions. 5d old WT (Col-0) seedlings were transferred to Glc free or 3% Glc containing ½ MS media and kept in light or dark for 3 d. Glc could cause root deviation even in absence of light ruling out the direct involvement of light in root directional growth. The time course study of **B,** gravitropic bending and **C,** root elongation in WT (Col-0) seedling roots after Glc treatments. 5d old uniformly grown seedlings were transferred to Glc free or increasing concentrations of Glc (1%, 3% and 5% Glc) containing ½ MS media and the direction of gravity was altered by turning the plates at 90º. Angle of root curvature and root length was measured after various time points. Delayed gravitropic bending was observed at increased concentrations of Glc (3%, 5% Glc), although root growth in 0%Glc vs. 5% Glc and 1% Glc vs. 3% Glc treated seedlings was comparable.

Data shown is the average of two biological replicates having atleast 30 seedlings; error bars represent SE; (Student' s t-test; P<0.001; * control vs. treatment).

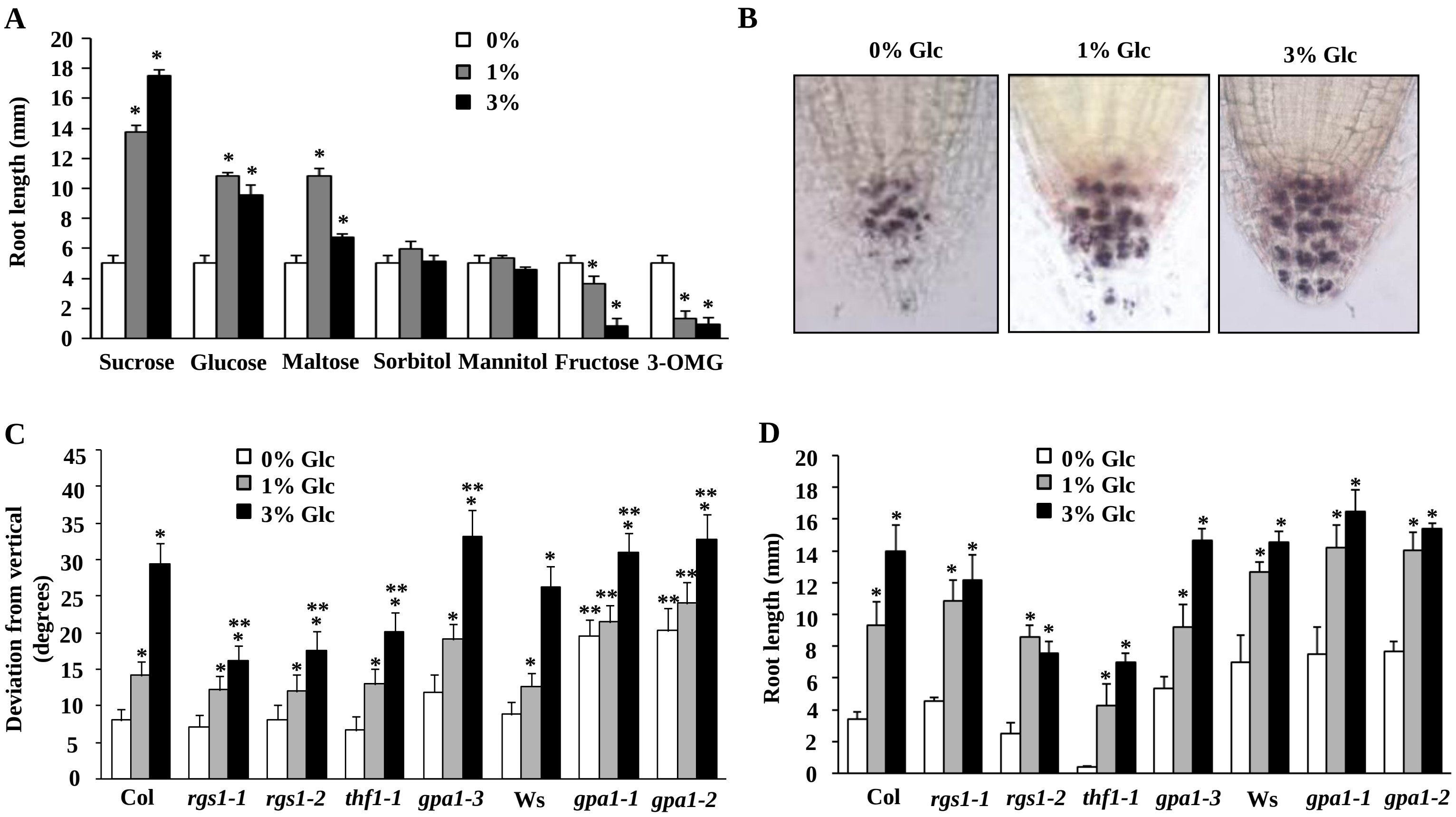

Supplementary figure S2. Role of Glc metabolism and signaling in root growth and direction.

A, Quantification of root elongation at indicated concentrations of various sugar analogs after 72h. **B,** I_2-KI staining of (Col-0) seedling root tip in presence of Glc free or increasing concentrations of Glc (1% and 3% Glc) containing ½ MS media. **C,** Analysis of Glc induced root deviation from vertical in HXK1-independent Glc signaling mutants. *rgs1-1*, *rgs1-2 and thf1-1* mutants displayed a significantly reduced response whereas the Glc induced root deviation was increased in *gpa1-1*, *gpa1-2 and gpa1-3* mutants. **D,** Quantification of root elongation in HXK1-independent Glc signaling mutants in Glc free or increasing concentrations of Glc (1% and 3% Glc) containing ½ MS media after 72 h.

Graphical data is the average of two biological replicates having atleast 30 seedlings; error bars represent SE; (Student's t-test; P<0.001; * control vs. treatment; ** WT vs. mutant).

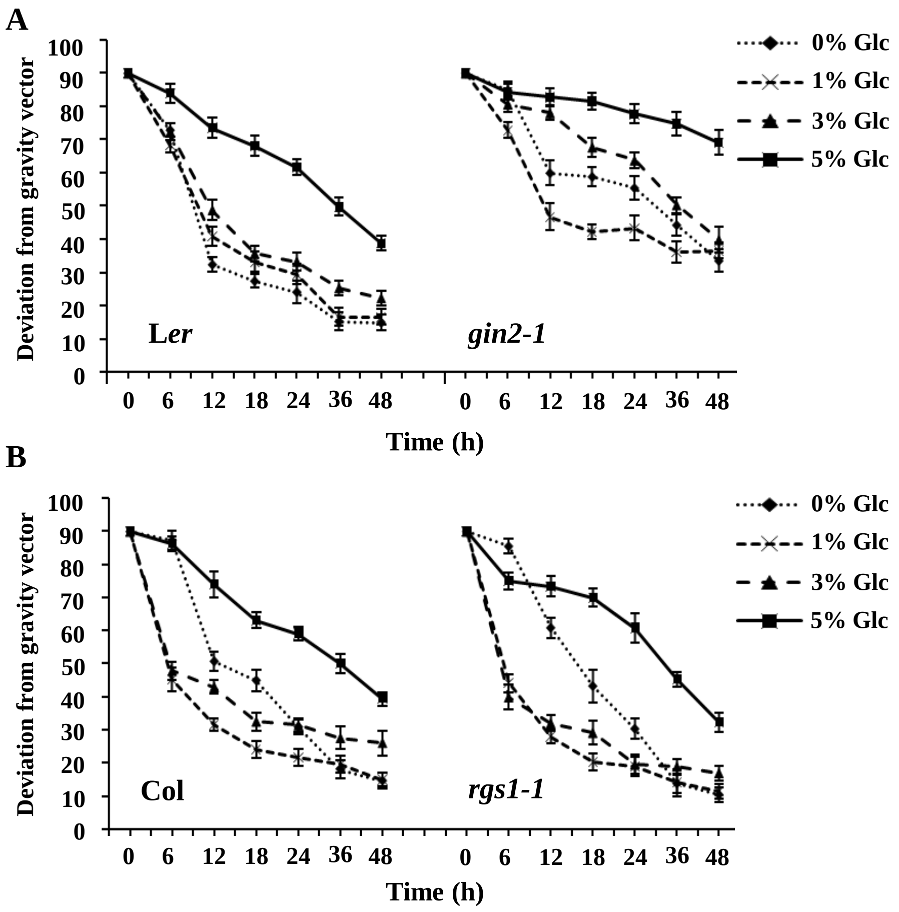

Supplementary figure S3. Both HXK -dependent and -independent components of Glc signaling are involved in root deviation from vertical response.

The time course of root curvature response in **A,** *gin2* and **B,** *rgs1-1*, mutants with respect to WT seedlings after 90^0 gravistimulation at indicated Glc concentrations. 5d old uniformly grown seedlings were transferred to Glc free or increasing concentrations of Glc (1%, 3% and 5% Glc) containing ½ MS media and the direction of gravity was altered by turning the plates at 90° . Angle of root curvature was measured after 6h, 12h, 18h, 24h, 36h and 48h of gravistimulation. The gravitropic growth was highly reduced in *gin2* mutant roots at any Glc concentration and time point.

Data shown is the average of two biological replicates having atleast 30 seedlings; error bars represent SE; (Student's t-test; P<0.001).

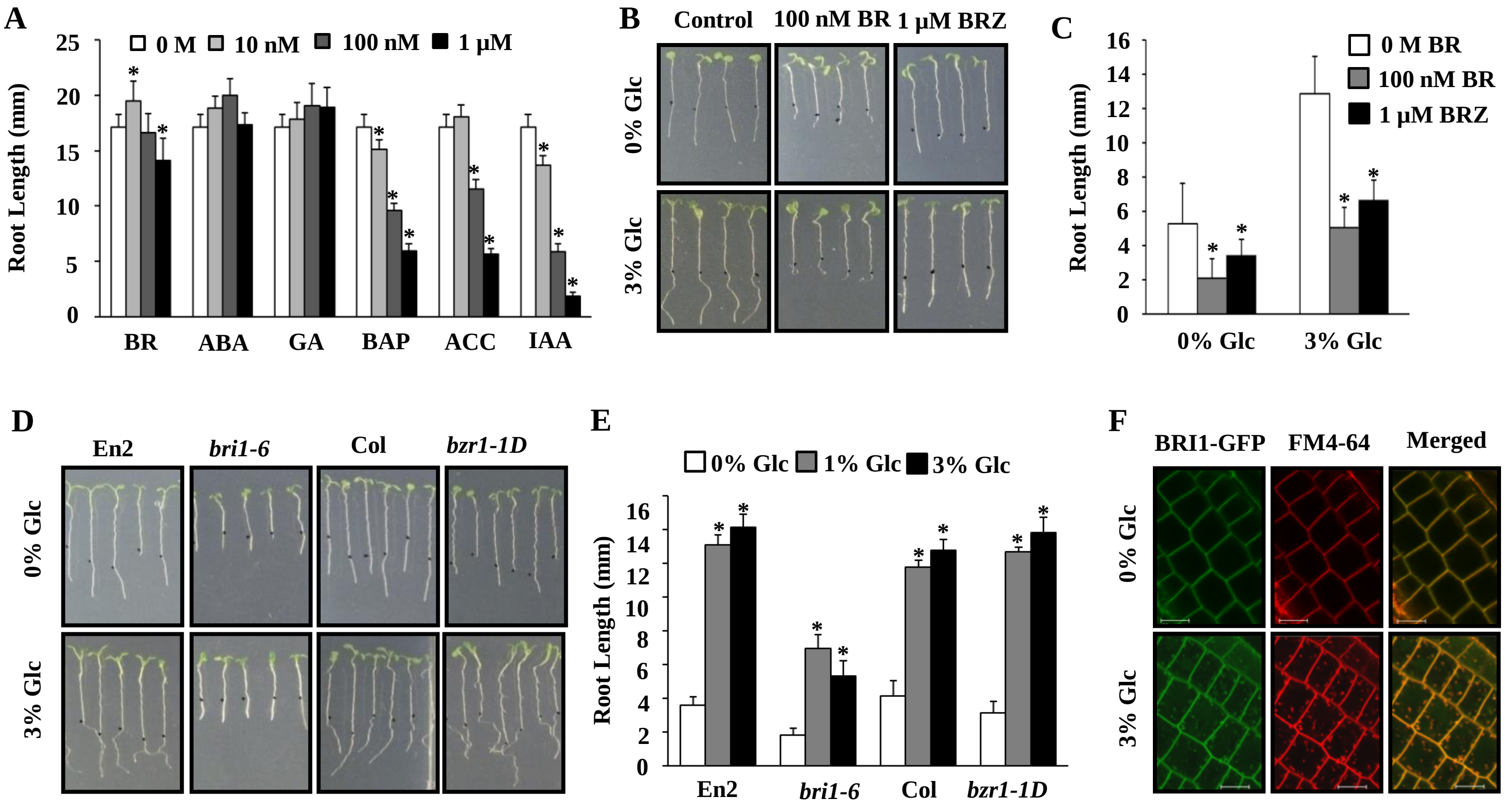

Supplementary figure S4. Involvement of BR-signaling elements in regulating root directional growth.

A, Quantification of root elongation of WT (Col-0) seedlings transferred to different phytohormones containing ½ MS media after 72 h. **B,** Pictures showing Glc-induced root deviation response of WT in absence or presence of 24-Epibrassinolide (BR) and BRZ. **C,** Quantification of root elongation of WT seedlings in Glc free or 3% Glc containing ½ MS media supplemented with or without BR or BRZ. **D,** Pictures showing Glc-induced root deviation response of WT and BR perception (*bri1-6*) and signaling (*bzr1-1D*) mutants. **E,** Quantification of root elongation in WT, *bri1-6* and *bzr1-1D* mutants in presence of Glc free or increasing Glc concentration (1%, 3%) containing ½ MS media. **F,** Increased BRI1 internalization to endosomal compartments of upon 3% Glc treatment as visualized via pBRI1::BRI1::GFP fluorescence and FM4-64 co-localization.

Graphical data is the average of two biological replicates having atleast 30 seedlings; error bars represent SE (Student's t-test; $P<0.05$; * control vs. treatment).

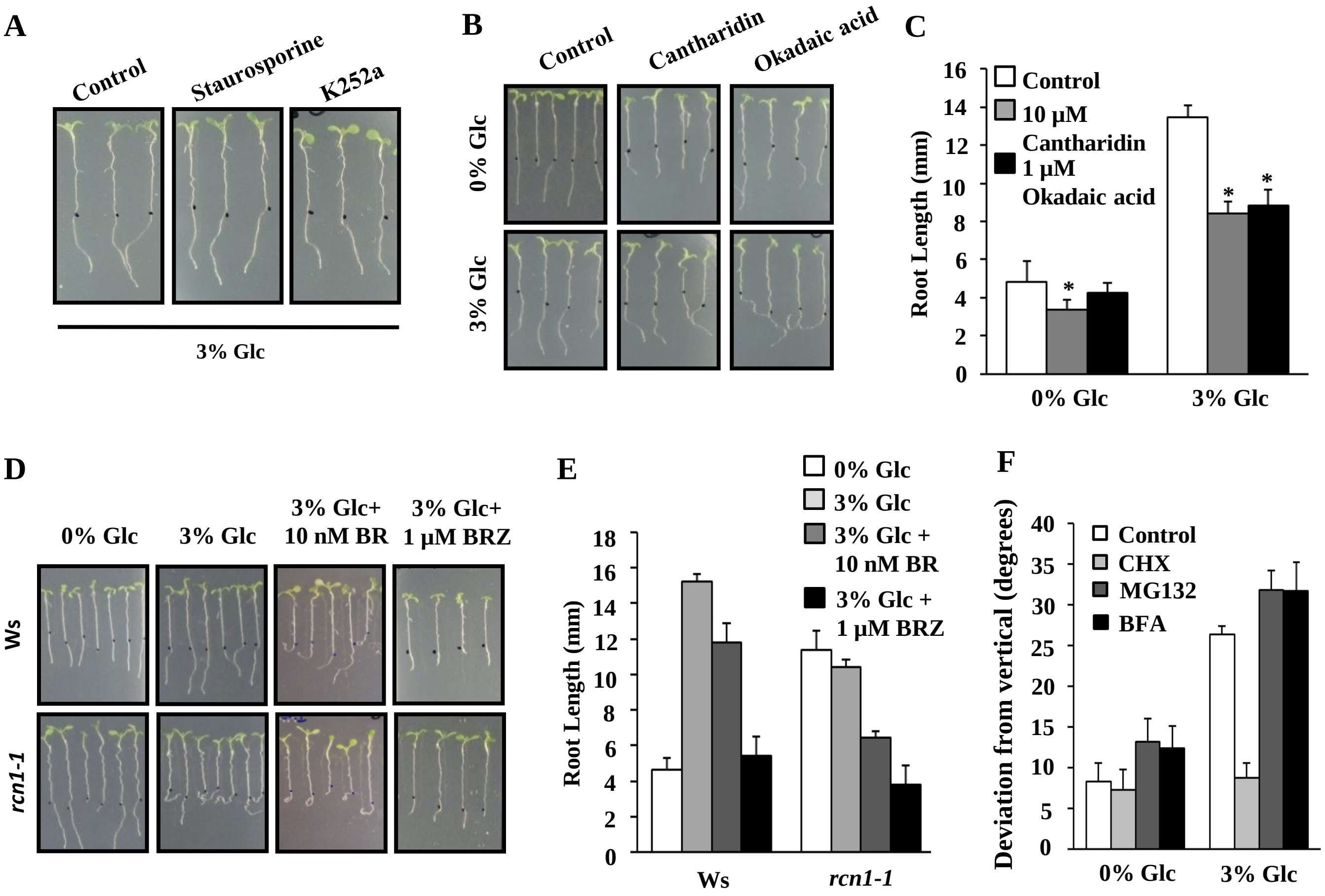

Supplementary figure S5. Involvement of protein phosphorylation and dephosphorylation in regulating Glc induced root directional response.

A, Pictures showing effect of protein kinase inhibitors staurosporine (500 nM) and K252a (100 nM) on Glc induced root deviation from vertical. **B,** Pictures showing enhanced Glc induced root deviation from vertical upon 1 µM okadaic acid and 10 µM cantharidin treatments. **C,** Quantification of root elongation in WT (Col-0) seedlings upon 1 µM okadaic acid and 10 µM cantharidin treatments in absence or presence of Glc. **D,** Pictures showing root directional growth and **E,** quantification of root elongation of WT and *rcn1-1* mutants on Glc free or 3% Glc containing media supplemented with or without 10 nM BR or 1 µM BRZ. **F,** Comparison of root directional growth of WT (Col-0) upon 1 µM CHX, 25µM MG132 and 5µM BFA treatments in presence and absence of Glc.

Graphical data is the average of two biological replicates having atleast 20 seedlings; error bars represent SE (Student's t-test; $P<0.001$; * control vs. treatment).

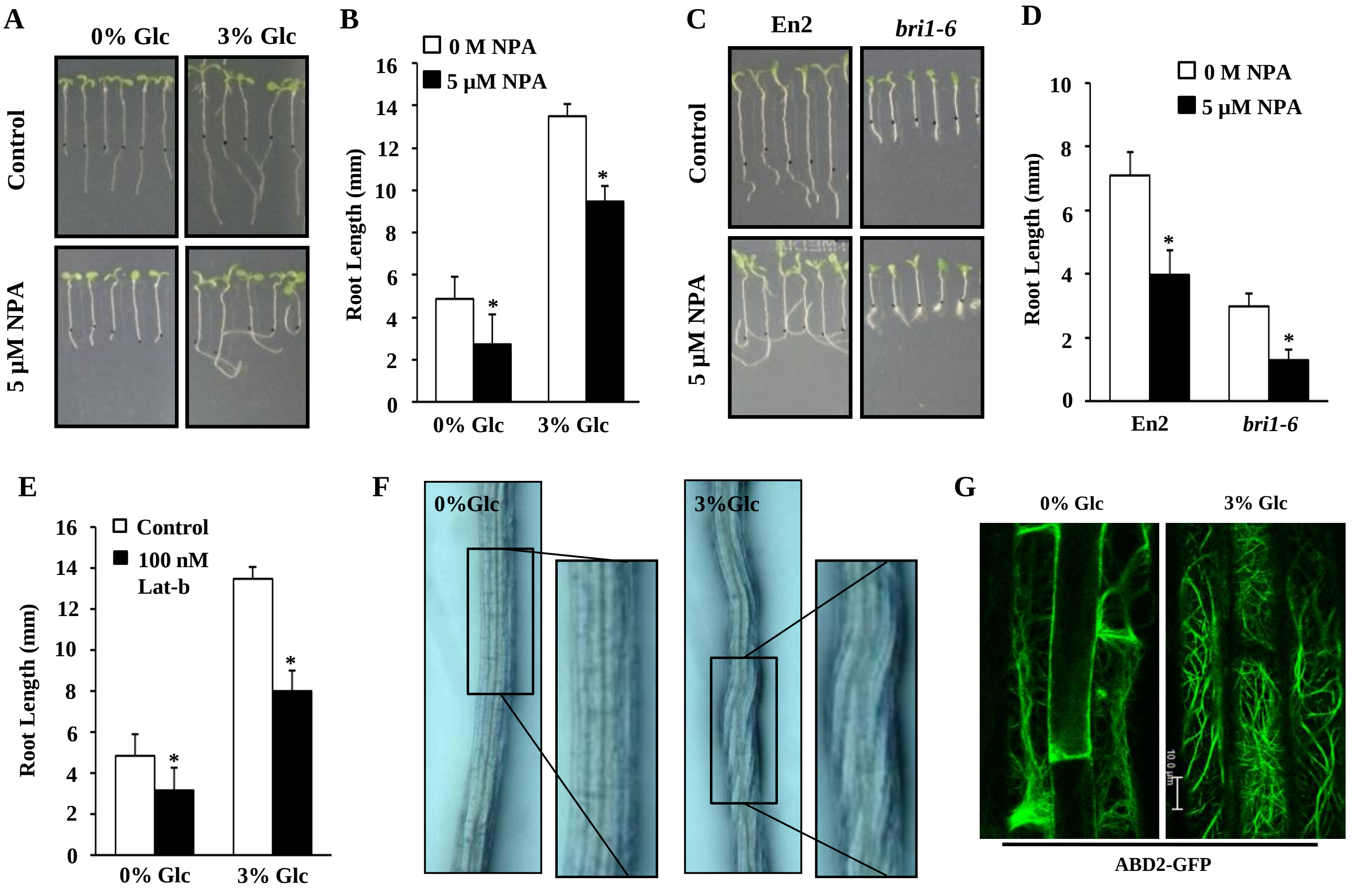

Supplementary figure S6. Changes in PAT and actin cytoskeleton organization regulates Glc induced root directional response.

A, Pictures showing **e**ffect of PAT inhibitor (NPA) on root deviation of WT (Col-0) seedlings. **B,** Quantification of root elongation in WT (Col-0) seedlings transferred to Glc free or 3% Glc containing ½ MS medium supplemented with or without NPA (5 μM). **C,** Pictures showing **e**ffect of PAT inhibitor (NPA) on root deviation of WT (En-2) and *bri1-6* mutant seedlings. NPA treatment along with 3% Glc could cause root deviation in *bri1-6* mutant which is resistant to Glc-induced root deviation response. **D,** Quantification of root elongation in WT and *bri1-6* mutant seedlings transferred to 3% Glc containing ½ MS medium supplemented with or without NPA (5 μM). **E,** Quantification of root elongation in WT (Col-0) seedlings transferred to Glc free or 3% Glc containing ½ MS medium supplemented with or without Lat-b (100 nM). **F,** Stereo-Zoom (Nikon SMZ1500) microscopic images showing the alignment of epidermal cell files. The epidermal cell patterning changes from straight profile to spiral upon Glc treatment. **G,** 35S::GFP-ABD2-GFP fluorescence in 5d old light-grown seedlings root tip, treated with 0% Glc and 3% Glc for 24 h. All confocal images were generated by using 63X objective (Scale bar 10.0 μM).

Graphical data is the average of two biological replicates having atleast 30 seedlings; error bars represent SE (Student's t-test; P<0.001; * control vs. treatment).

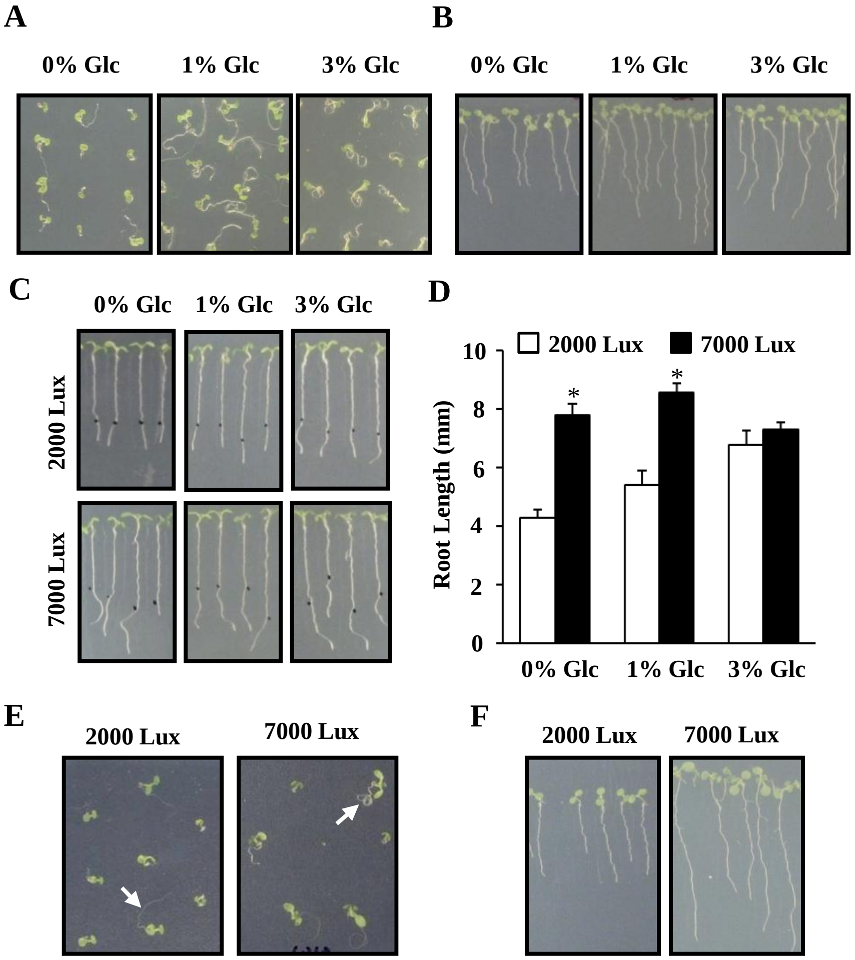

Supplementary figure S7. High light intensity could mimic Glc effects on root waving and coiling responses.

A, Root-coiling and **B,** waving phenotypes of WT (Col-0) seedlings in absence (0% Glc) or presence of increasing Glc concentrations (1%, 3% Glc). **C,** Pictures showing root deviation from vertical in WT (Col-0) seedlings at low (2000 Lux) and high (7000 Lux) light intensities. **D,** Quantification of root elongation in WT (Col-0) seedlings transferred to ½ MS media at low (2000 Lux) and high (7000 Lux) light intensities. **E,** Root coiling and **F,** root waving response of WT (Col-0) seedlings at low (2000 Lux) and high (7000 Lux) light intensities.

Graphical data is the average of two biological replicates having atleast 15 seedlings; error bars represent SE (Student's t-test; P<0.001; * control vs. treatment).

S.no.	Gene name	Representative Public ID	Forward (5ʹ ---- 3ʹ)	Reverse (5ʹ ---- 3ʹ)
**1.**	**CPD**	**AT5G05690**	CCATTGAAGCAGAAGAGATTTATGC	AAAGCCTTAGCTTCTGTGTACATAAAAA
**2.**	**DWF4**	**AT3G50660**	GCATCATACTCTCTTACCTCTTCTT	CCAAATTTTTATATATCATTGGGCA
**3.**	**ROT3**	**AT4G36380**	TGTCATGTCAAATTATAAGCGTTGGT	TCAACTTGCGTTTGGTACGATAAA
**4.**	**BR6OX2**	**AT3G30180**	TCTCTCTTTCTCTCTGTACATTATCCATCT	AGGGTCATTTTGTAGGGCTAATTAAC

Supplementary Table S1. List of primers used for real-time PCR analysis.
